# The Efficacy of Virtual Fracture Clinics (VFCs) and the Impact of Physician Risk Appetite on Discharge Rates

**DOI:** 10.7759/cureus.51798

**Published:** 2024-01-07

**Authors:** Abhirun Das, Jie Xi Tan, Anand Pillai

**Affiliations:** 1 Trauma and Orthopaedics, Wythenshawe Hospital, Manchester University National Health Service (NHS) Foundation Trust, Manchester, GBR; 2 Trauma and Orthopaedics, University of Manchester, Manchester, GBR

**Keywords:** discharge, lower limb, upper limb, risk appetite, virtual fracture clinic

## Abstract

Background and objectives

Virtual fracture clinics (VFCs) allow the triage of emergency department referrals to identify those requiring further care and those that are suitable for discharge. Appropriate discharge from VFC benefits the patient and the healthcare provider by avoiding unnecessary face-to-face appointments. This study investigates factors associated with VFC discharge rates at our hospital and detects potential areas for improvement.

Methodology

A retrospective review was conducted on 4819 consecutive VFC referrals between March 17, 2021, and March 16, 2022, from a single hospital. Patient demographics, referral outcomes, and triaging consultant data were collected. Sixteen consultants conducted daily VFCs during the study period. Eleven consultants completed the DOSPERT psychometric test to measure their attitude toward risk. The data was analysed using Spearman's rho and Chi-square tests.

Results

The mean discharge from VFC was 35.4% (29.6-41.0%). The highest rates of discharge were for back pain (100%), followed by fractures of the pubic ramus (100%), the base of the fifth metatarsal (86.89%), the acetabulum (75%), and the proximal radius (73.03%). Consultant experience was significantly negatively correlated with discharge rate (p<0.05). The frequency of conducting a VFC was not associated with the discharge rate (p=0.758). In subspecialty analysis, 90% of lower limb consultants discharged more lower limb presentations from VFC, compared with upper limb consultants (p=0.001). There was no significant correlation between DOSPERT scores and discharge rates (p=0.65).

Conclusions

VFC remains an important tool for patient care. Consultant experience is associated with a more cautious approach to discharge; however, there was no relationship between a consultant’s risk attitude and their VFC discharge rate in this study. Lower-limb consultants appear to discharge lower-limb injuries more readily when compared with their upper-limb colleagues. These insights could be used to improve emergency department and VFC efficiency.

## Introduction

The virtual fracture clinic (VF) care model originated from Glasgow Royal Infirmary in 2011 and aimed to standardise routes for treatment whilst also finding ways to optimise resource allocation. VFC was adopted at our institution in August 2015 and is used to triage non-urgent referrals from general practice and the emergency department. There are guidelines in place in our institution to aid physician decision-making regarding appropriate VFC referrals [1].

VFCs are held daily with a designated consultant trauma and orthopaedic surgeon and a trauma specialist nurse. Patient notes, referral letters, and digital imaging on picture archiving and communication systems (PACS) are reviewed. Each patient receives a decision: discharge with an information leaflet and advice, follow-up (clinical follow-up, physiotherapy follow-up, plaster room follow-up), or urgent review, and these are communicated to the patient or referring GP [2,3].

Previous studies on VFC have shown numerous benefits for the health service. It is a safe form of patient care [4,5]. When compared to traditional face-to-face review for all referrals, it is more cost-efficient for both the provider [6,7] and the patient [8]. It is also more time efficient as each referral is reduced from an 11-minute face-to-face review to a one-minute virtual review [9] which allows for more efficient use of the multidisciplinary team’s time [8,10]. Importantly, it also has a high level of patient satisfaction [6,11] and was particularly useful for social distancing during the COVID-19 pandemic [12-15].

This study investigates the effectiveness of VFC at our institution and explores the role of physician experience, sub-specialty, and risk appetite in the rate of discharged referrals.

## Materials and methods

Prior approval was obtained from the local audit department. A retrospective review of 4822 consecutive VFC referrals at Wythenshawe Hospital, Manchester, United Kingdom, between March 17, 2021, and March 16, 2022, (a duration of one year) from the E-trauma platform was extracted and structured into an Excel worksheet (Microsoft Corporation, Washington, USA), which was then formatted for analysis. The following information was collected: VFC date, age, gender, consultant, diagnosis/diagnoses, and outcome. Further details about each referral were taken from the Electronic Patient Record (EPR) (E-trauma). Sectra PACS was used to review any diagnoses by the consultant in charge of the VFC referrals for that day if they were not clear from the VFC outcome.

Every VFC referral at Wythenshawe Hospital within the above-mentioned period was included in the study. Two referrals were excluded as they were self-discharged to another hospital. Over the study period, one consultant performed one assessment and was thus excluded from the analysis to avoid confounding. Following the exclusion, a total of 4819 patient records were included in this study’s dataset.

In the raw database from E-trauma, the referral outcome 'outcome' was classified as either 'clinical follow-up', 'plaster room follow-up', or 'physiotherapy follow-up'. These were all grouped as 'follow-up' to ensure the data was categorised appropriately for analysis. Additionally, each patient’s diagnosis was classified as either upper limb (UL), lower limb (LL), or foot and ankle, and the total number of diagnoses presented by each patient was calculated. Information regarding the specialty of each consultant (classified UL and LL) and the experience (in years) as a consultant were obtained for further analysis.

The data was analysed using SPSS Statistics (IIBM Corp. IBM SPSS Statistics for Windows, Armonk, NY: IBM Corp.), and descriptive data of variables (VFC date, gender, age, consultant, UL/LL, number of diagnoses, and outcome) were generated to give insights into and validate the reliability of the dataset. The variables were cross-tabulated to determine their significance.

## Results

The mean age of referred patients was 39 years old (range 1-99 years). On average, 49% of patients were male, and 51% were female. Analysing with respect to the time period, the busiest quarter can be identified as Q2 (n=1407), with the busiest month recorded as April (n=482). On average, the busiest day of the week in terms of the most number of assessments conducted on a specific day was recorded as Thursday (n=812) with Sunday (n=576) recording the lowest number of assessments conducted.

There were 16 consultants, six ULs, and 10 LLs (hip, knee, foot, and ankle). The mean number of consultant assessments was 301 (range 30-641). About 2948 (61.1%) assessments were for UL injuries, and 1868 (38.8%) were for LLs. Three referrals were for both UL and LL injuries.

In total, there were 4560 (94.6%) assessments with one diagnosis, 246 (5.1%) with two, 13 (0.27%) with three, and one (0.02%) with four diagnoses. There were 1344 (27.89%) assessments for paediatric patients (<16 years old), with 641 (47.7%) discharged, 671 (49.9%) followed up, and 32 (2.3%) urgently reviewed. Overall, 1704 (35.36%) patients were discharged, 2883 (59.83%) were scheduled for follow-up, and 232 (4.81%) were scheduled for urgent review. The most common diagnoses per outcome category (discharged, followed up, and urgently reviewed) were identified, as can be seen in Table 1 below.

**Table 1:** Top 3 most commonly discharged, follow-up, and urgently reviewed diagnoses

Discharged cases	Number of discharges (n)	Follow-up cases	Number of follow-ups (n)	Urgent review cases	Number of urgent review cases (n)
Injury of ankle	105	Fracture of distal end of radius	422	Fracture of distal end of radius	77
Fracture of lateral malleolus	98	Fracture of scaphoid bone of wrist	330	Fracture of ankle	12
Fracture of radial head	98	Dislocation of shoulder joint	122	Rupture of Achilles tendon	8

The top five diagnoses that were discharged from the VFC are shown in Table 2: back pain (n = 3) with 100% got discharged; fracture of pubic rami (n=3) with 100% discharged; base of fifth metatarsal fractures (n=122) with 86.89% discharged; fracture of acetabulum (n=4) with 75% discharged; and fracture of proximal radius (n=178) with 73.03% discharged.

**Table 2:** Top 5 commonly discharged diagnoses (in percentage) from VFC

Diagnosis	Number of discharge	Total presentation	Percentage of discharge
Back pain	03	03	100.00%
Fracture of pubic rami	03	03	100.00%
Fracture of acetabulum	03	04	75.00%
Fracture of fifth metatarsal (base)	106	122	86.89%
Fracture of radius (proximal)	130	178	73.03%

Table 3 shows that a total of 4539 patients presented with one diagnosis (with a discharge rate of 36.07%), 265 with two (discharge rate of 24.91%), 14 with three (discharge rate of 7.14%), and only one with four (0.00%). An independent sample test was used to assess the significance between the number of diagnoses and the outcome, which shows that the result is significant (p<0.001). This result showed that the percentage of discharge decreased with the increased number of diagnoses.

**Table 3:** Outcome by number of diagnoses per assessment

Number of diagnoses	Number of follow-up	Number of discharge	Total assessment	Percentage of discharge
1	2902	1637	4539	36.07%
2	199	66	265	24.91%
3	13	1	14	7.14%
4	1	0	1	0.00%
Total	3115	1704	4819	

**Figure 1:**
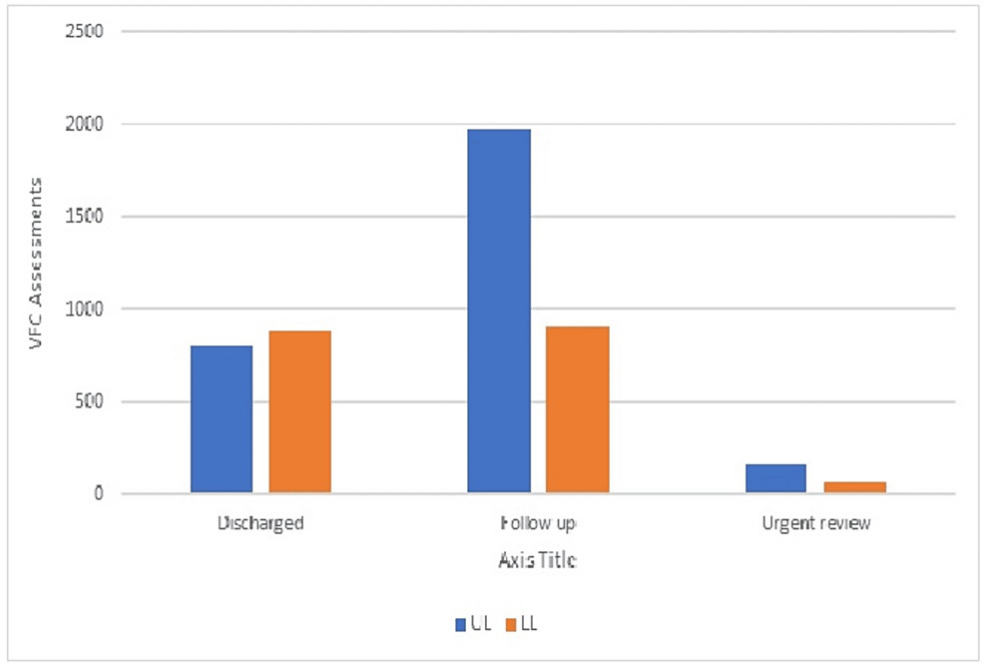
VFC assessments of UL/LL by outcome UL: upper limb, LL: lower limb, VFC: virtual fracture clinic

There were a total of 2948 UL assessments, 1868 LL assessments, and three assessments with both UL and LL needing to be updated for FA. The percentage of discharge for LL is 52.4% in comparison to 47.5% for UL. The statistical significance was calculated using the Chi-square test, showing that the result is significant (p<0.001). This showed that there is a significantly higher number of patients presented with UL than LL and a higher percentage of discharge for LL assessments, as shown in Figure 1 below.

Table 4 shows all assessments broken down by outcome at a consultant level. Spearman’s rho was used to assess the correlation between the percentage of discharge and total assessments for a consultant. This found that the findings were not significant (p=0.758).

**Table 4:** Total assessment breakdown per consultant

Consultant	Follow-up	Discharge	Urgent review	Total	Percentage of discharge
Consultant A	132	91	9	232	39.22%
Consultant B	199	111	12	322	34.47%
Consultant C	178	104	19	301	34.55%
Consultant D	169	123	8	300	41.00%
Consultant E	245	149	25	419	35.56%
Consultant F	102	60	6	168	35.71%
Consultant G	397	219	25	641	34.17%
Consultant H	118	72	13	203	35.47%
Consultant I	121	56	12	189	29.63%
Consultant J	32	19	1	52	36.54%
Consultant K	225	144	26	395	36.46%
Consultant L	329	219	19	567	38.62%
Consultant M	273	115	16	404	28.47%
Consultant N	215	143	19	377	37.93%
Consultant O	131	68	20	219	31.05%
Consultant P	17	11	2	30	36.67%
Total	2883	1704	232		

The consultants were grouped based on their experience (in years) as consultants into the following categories (Table 5).

**Table 5:** Grouping of consultants based on their years of experience into categories

Category	Years of experience
1	Consultants with one to five years of experience
2	Consultants with six to ten years of experience
3	Consultants with eleven to fifteen years of experience
4	Consultants with twenty or more years of experience

An independent sample test was used to assess the correlation between the years of experience of the consultant and the outcome, which shows that the result is significant (p<0.05). This result indicated that the consultants who have more experience (in terms of years) have a lower likelihood of discharging a patient (Table 6).

**Table 6:** Consultant experience (in years) with respect to percentage of discharges

Consultant	Experience (in years)	Category	Percentage of discharge
Consultant A	2	1	39.22%
Consultant B	15	3	34.47%
Consultant C	1	1	34.55%
Consultant D	3	1	41.00%
Consultant E	10	2	35.56%
Consultant F	5	1	35.71%
Consultant G	10	2	34.17%
Consultant H	15	3	35.47%
Consultant I	20	4	29.63%
Consultant J	20	4	36.54%
Consultant K	10	2	36.46%
Consultant L	15	3	38.62%
Consultant M	12	3	28.47%
Consultant N	3	1	37.93%
Consultant O	14	3	31.05%
Consultant P	1	1	36.67%

Consultants were labelled as ‘agree’ when there was a higher percentage of discharge for assessments within their specialty as compared to the alternative category, whereas consultants were labelled as ‘disagree’ when there was a lower percentage of discharge for assessments within their specialty relative to the alternative category.

With regards to the ‘agree’ category, 90% of all consultants who specialised in LL agreed with the above, and 0% specialised in UL agreed. The Chi-square test performed indicated that this is statistically significant (p=0.001).

With regards to the ‘disagree’ category, 10% of consultants who specialised in LL disagreed with the above and 100% specialised in UL disagreed. The Chi-square test performed indicated that this is statistically significant (p=0.001).

## Discussion

The results show that the busiest day of the week was Thursday, whilst the busiest quarter was Q2 (April to June), with April being the busiest month. This data could inform fracture clinic resource allocation to cope with the peak levels of demand. It was also found that there were significantly more assessments with UL than LL presented to the VFC. Back pain is an inappropriate referral to VFC and should be directed to the musculoskeletal referral network through their general practitioner if there is no acute injury identified [3].

However, other hospitals, such as Royal Sussex Hospital in Brighton, do explicitly have a back pain management protocol that mentions that patients should not be referred to the VFC; rather, they should be reviewed by a GP or referred to spine service as an outpatient after excluding any potential infections [16]. It may be of value to adopt this framework at our hospital to reduce our patients' need to attend their general practitioner for review and referral.

The findings show that the discharge rate for fracture of the proximal radius was 73.03%. Local policy states that undisplaced/minimally displaced fractures should be discharged straight from the ED with advice and analgesia [17]. However, findings show that there were a significant number of patients with this 'undisplaced/minimally displaced' radial head/neck fracture who were referred to the VFC and were discharged from the VFC without clinical follow-up required [17]. This may be due to diagnostic uncertainty or a lack of confidence amongst our emergency department colleagues and is a potential area for improvement in referrals, clinical administration burden, and patient care.

The results show that the discharge rate for the base of the fifth metatarsal fracture was 86.9%. Local guidelines state that all bases of fifth metatarsal fractures should be referred to VFC. Other hospitals take a different approach, whereby patients are provided with an ‘elasticated bandage’ or 'removable boot’ and an information leaflet in the ED [18] unless there is diagnostic uncertainty and therefore can be referred to VFC. This method has been shown to be reliable, successful, and patient-focused in discharging patients with a base of fifth metatarsal fracture from the ED [18].

The discharge rates for the fracture of the public rami and the fracture of the acetabulum were 100% and 75%, respectively. With respect to the BOAST guidelines, pelvic fractures should have a defined referral pathway and be managed via a trauma system due to potential side injuries such as major haemorrhage and urological injuries [19]. However, this guidance only applies to pelvic ring fractures, whereas isolated acetabular fractures and isolated low-energy pubic rami fractures are not included [19]. Therefore, the results show that the BOAST guidelines are being adhered to for these fractures. Whilst the VFC consultants are adhering to the BOAST guideline to discharge patients with acetabular or pubic rami fractures, the VFC referral guidelines can be updated to reflect the BOAST guidance and prevent the initial referral.

The data observed identifies a significant correlation between the number of diagnoses and the rate of discharge. The greater the number of diagnoses, the more likely clinical follow-up is required. This indicates that patients with more injuries/fractures present more complex cases, as there are more factors to consider. Therefore, it means that ED can be more confident in discharging patients with only one diagnosis compared to patients presenting with multiple diagnoses.

It was found that UL presentations had a lower discharge outcome, whereas LL presentations had a higher discharge outcome. The most followed-up were fractures of the distal radius and scaphoid. The total number of assessments for each referral diagnosis was 583 and 344, respectively.

For fractures of the distal end of the radius, 90.7% of assessments were followed up in total, with 84.3% non-urgent reviews and 15.7% urgent reviews. For the fracture of the scaphoid bone, 97.6% of assessments were followed up in total, with 98.2% of assessments non-urgently reviewed and 1.8% of assessments urgently reviewed.

A fracture of the distal radius will need to be followed up, regardless of displacement, for either an operation or the removal of a cast after six weeks [20]. Patients presented with fractures of the scaphoid bone will need to be followed up for orthopaedic review with delayed radiographs, or MRI, to detect an occult fracture and prevent severe complications associated with avascular necrosis [21]. Given the significant number of assessments with these UL presentations, it is unsurprising that the discharge rate is lower for UL presentations compared to LL presentations.

Increase discharge from VFC

Consultant experience was analysed as a measure in two ways: total assessments performed in VFC and years of experience as a consultant. The findings showed that consultants who performed more VFC assessments did not necessarily discharge more patients. This might be because the type of assessments performed varies across all consultants, which implies that these assessments cannot be treated homogenously and are not a valid measure to assess discharge correlation.

Additionally, the result showed that consultants who have more experience (in years) tend to discharge fewer patients. The lower discharge rate could be explained by senior consultants leveraging their experience to identify patterns for potential issues that may require follow-up appointments to analyse in detail. This may suggest that senior consultants take a more cautious approach, given their range of experience.

Consultant subspecialty

The results showed that for UL presentations, consultants who specialised in UL were not more likely to discharge patients with UL presentations; instead, all UL consultants were more likely to discharge patients with LL presentations. For LL presentation, the result proved significant, as 90% of LL-specialised consultants discharged more LL-presented patients than UL.

With both UL-specialised consultants and LL-specialised consultants discharging more LL-presented patients, it shows that the specialty of consultants does not necessarily have an impact on which type (UL/LL) assessments they discharge. However, this finding might indicate that the nature of UL presentations (referred to as VFC) is generally more complex in rehabilitation than LL presentations. Therefore, UL presentations have a higher likelihood of clinical follow-up than LL presentations.

Limitations

This study analysed practices at one hospital. Given the volume of referrals, this study did not consider the mechanism of injury, the degree of fracture displacement, or the outcome of those injuries that were discharged from VFC.

## Conclusions

The study results revealed a noteworthy correlation between consultants' experience with VFC referrals and their likelihood of discharging patients. Interestingly, consultants with greater experience demonstrated a tendency towards a more cautious approach, being less inclined to discharge patients compared to their less-experienced counterparts. Furthermore, regardless of subspecialty, consultants were more likely to discharge patients with LL presentations than those with UL presentations, leading to an increased need for clinical follow-ups in the UL cases. Additionally, consultants showed a lower likelihood of discharging patients with multiple diagnoses, underscoring the complexity involved in such cases.

The VFC is a reliable, effective, and efficient service that has proven to be a useful method that has benefited the NHS and patients alike. By scrutinising VFC activity, healthcare professionals can enhance the delivery of the service. Our data highlights opportunities for improving our VFC process through clinician education and opportunities to optimise resource allocation at our hospital. Future work could incorporate other acute sites within our trust.
